# Patterns of drug prescriptions in an orthogeriatric ward as compared to orthopaedic ward: results from the Trondheim Hip Fracture Trial—a randomised clinical trial

**DOI:** 10.1007/s00228-017-2263-x

**Published:** 2017-05-26

**Authors:** Marianne Heltne, Ingvild Saltvedt, Stian Lydersen, Anders Prestmo, Olav Sletvold, Olav Spigset

**Affiliations:** 1 Department of Health and Welfare Services, City of Trondheim, Trondheim, Norway; 2 Department of Neuromedicine and Movement Science, Faculty of Medicine and Health Sciences, Norwegian University of Science and Technology (NTNU), Trondheim, Norway; 3 Department of Geriatrics, St. Olav University Hospital, Trondheim, Norway; 4 Regional Centre for Child and Youth Mental Health and Child Welfare, Faculty of Medicine and Health Sciences, Norwegian University of Science and Technology (NTNU), Trondheim, Norway; 5 Department of Clinical Pharmacology, St. Olav University Hospital, Trondheim, Norway; 6 Department of Laboratory Medicine, Children’s and Women’s Health, Faculty of Medicine and Health Sciences, Norwegian University of Science and Technology (NTNU), Trondheim, Norway

**Keywords:** Geriatric assessment, Hip fracture, Pharmacotherapy, Comprehensive geriatric care, Polypharmacy, Frail elderly

## Abstract

**Purpose:**

In the Trondheim Hip Fracture Trial, 397 home-dwelling patients with hip fractures were randomised to comprehensive geriatric care (CGC) in a geriatric ward or traditional orthopaedic care (OC). Patients in the CGC group had significantly better mobility and function 4 months after discharge. This study explores group differences in drug prescribing and possible associations with the outcomes in the main study.

**Methods:**

Drugs prescribed at admission and discharge were registered from hospital records. Mobility, function, fear of falling and quality of life were assessed using specific rating scales. Linear regression was used to analyse association between drug changes and outcomes at 4 months.

**Results:**

The mean age was 83 years, and 74% were females. The mean number (± SD) of drugs in the CGC and OC groups was 3.8 (2.8) and 3.9 (2.8) at inclusion and 7.1 (2.8) and 6.2 (3.0) at discharge, respectively (*p* = 0.003). The total number of withdrawals was 209 and 82 in the CGC and OC groups, respectively (*p* < 0.0001), and the number of starts was 844 and 526, respectively (*p* < 0.0001). A significant negative association was found between the number of drug changes during the hospital stay and mobility and function 4 months later in both groups. However, this association disappeared when adjusting for baseline function and comorbidities.

**Conclusion:**

These secondary analyses suggest that there are significant differences in the pharmacological treatment between geriatric and orthopaedic wards, but these differences could not explain the beneficial effect of CGC in the Trondheim Hip Fracture Trial.

**Electronic supplementary material:**

The online version of this article (doi:10.1007/s00228-017-2263-x) contains supplementary material, which is available to authorized users.

## Introduction

Patients with hip fractures are often frail, of advanced age and with extensive comorbidity. Most patients suffering a hip fracture have osteoporosis, increased risk of falling and various disabilities. After a hip fracture, the prognosis is generally poor with high mortality and deteriorated functional status [1–3].

Pharmacotherapy in patients with hip fractures is challenging due to the patients’ extensive comorbidity and associated high frequency of polypharmacy even before the fracture [3, 4]. Moreover, about 50% of the patients develop acute delirium during the hospital stay. The risks of adverse drug reactions and other complications in the postoperative period are extensive because these patients are frail and particularly vulnerable and sensitive to adverse drug reactions.

Comprehensive geriatric care (CGC) is a multidimensional, interdisciplinary diagnostic and therapeutic process focusing on the medical, psychosocial, functional and social capabilities and limitations of frail elderly patients in order to improve health and quality of life [5]. CGC has been shown to improve outcomes for frail elderly in hospitals [6, 7]. Evaluation of pharmacological treatment with respect to indication, adverse effects, inappropriate prescribing and drug interactions is an essential part of CGC. In a randomised clinical trial, we have previously shown that patients receiving CGC in a geriatric ward had a more appropriate drug profile at discharge as compared with treatment in a general medical ward [8]. In a non-randomised study, Schmader et al. showed that by treating patients in a geriatric ward and outpatient clinic, serious adverse drug reactions and suboptimal prescribing were reduced, and underuse of drugs was identified [9].

The Trondheim Hip Fracture Trial is a randomised clinical trial where elderly home-dwelling patients with hip fractures at admission to hospital were randomised to receive CGC in a geriatric ward located in a medical department or usual orthopaedic care (OC) in an orthopaedic trauma ward. In the primary publication [10], we showed that CGC had significant beneficial impacts on mobility, function, quality of life and number of days in institutions, while also being cost-effective. As part of the CGC, a comprehensive medical assessment and a review of each patient’s drug regimen with focus on appropriate pharmacological treatment and screening for vitamin deficiencies, urinary tract infections, pain, constipation, falls, orthostatic hypotension and osteoporosis were performed [11].

CGC has often been described as a “black box” consisting of many elements that are often not easy to describe in detail. The present study is based on post hoc analyses; the specific aim was to investigate how drug treatment provided as part of the CGC differed from that offered in traditional orthopaedic care and whether differences in drug prescribing can explain the beneficial impact of CGC on mobility, function, quality of life and fear of falling 4 months after discharge from hospital, as shown in our primary publication [10]. More specifically, we wanted to study the number of drugs prescribed per patient, the extent of polypharmacy, the prescription of drugs with potential anticholinergic adverse effects and the extent and type of changes in drug prescription between admission and discharge. To avoid falls, there was focus on cardiovascular drugs and drugs acting on the nervous system, and prescribing patterns of these drugs were therefore studied in detail. The CGC treatment protocol had specific focus on drug therapy for osteoporosis, constipation, pain and nutrition [11].

## Material and methods

**Fig. 1:**
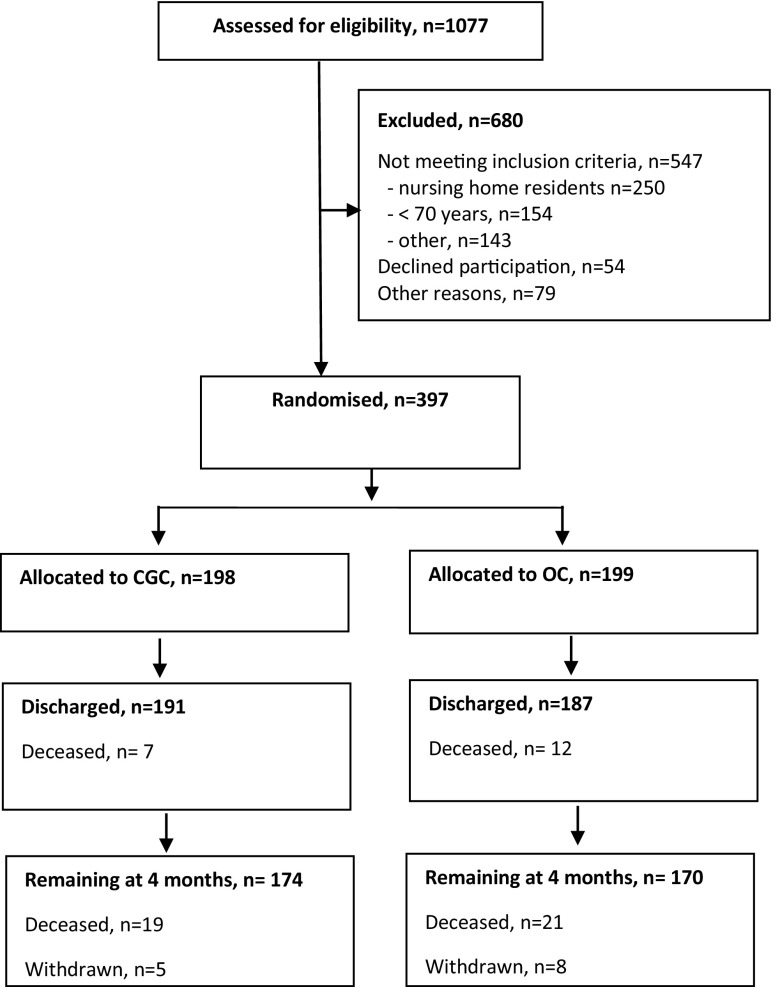
Patient flow. Patients admitted to the hospital with hip fractures were screened in the emergency department and randomised after giving their informed consent. *CGC* comprehensive geriatric care, *OC* orthopaedic care

The present study is a substudy of the Trondheim Hip Fracture Trial that is a single-centre, prospective randomised clinical trial performed at St. Olav University Hospital in Trondheim, Norway. Patients were recruited between 17 April 2008 and 30 December 2010. The protocol, treatment program and other outcomes have been published elsewhere [10–15]. Patient flow is shown in Fig. 1.

### Study design

Home-dwelling patients with hip fractures aged 70 years and older who had been able to walk 10 m or more prior to the hip fracture were eligible for inclusion. Patients with pathological fractures, multiple trauma and short life expectancy; those living permanently in nursing homes; or those already participating were excluded.

After the orthopaedic surgeon had diagnosed a fracture, the patients gave their informed consent to be included in the study. If a patient was too cognitively impaired to be asked, his or her next of kin gave this consent. The patients were randomised by the nurses in the emergency room to receive either CGC or OC and were thereafter transferred directly to their allocated ward. Participants were randomised in a ratio of 1:1, and the random sequence was computer-generated in blocks of a size unknown to the investigators. A Web-based computer-generated service prepared by the Unit of Applied Clinical Research, Norwegian University of Science and Technology (NTNU) was applied.

### Medical treatment during the trial

Details on treatment in the two groups are shown in Supplementary Table 3. Geriatricians, also being specialists in internal medicine, were responsible for medical treatment in the CGC group whereas orthopaedic surgeons were responsible in the OC group. If needed, other specialists were consulted to give advice on medical treatment. All patients received perioperative prophylaxis with antibiotics and a 2-week postoperative prophylactic treatment against venous thromboembolism. After discharge from hospital, general practitioners or physicians at the rehabilitation facilities treated patients in both groups.

### In the geriatric ward

In the CGC treatment arm, a comprehensive assessment of each patient’s somatic and mental health was completed, based on assessment and observations of patients during the hospital stay. In addition, information on pre-fracture status was collected from medical records and by interviewing patients and caregivers. All patients were screened for urinary tract infections and underwent repeated blood pressure measurements, including orthostatic blood pressure, and a comprehensive blood screen, including electrolytes, creatinine and estimated glomerular filtration rate, vitamin B_12_ and folate. Vitamin D was analysed in selected patients. The drug regimen was evaluated for each patient regarding indication for use, potential adverse effects and doses, in line with recommendations for prescribing in frail elderly patients. The geriatric ward had fortnightly teaching sessions focusing on appropriate drug treatment in close collaboration with clinical pharmacologists, including the use of the Anticholinergic Risk Scale (ARS) and the STOPP and START criteria [16–18], although these criteria were not applied systematically to each CGC patient. If indicated, treatment for recently diagnosed disorders was initiated during the hospital stay.

Patients with hip fractures are frequently cognitively impaired, and in most patients, the hip fracture is a consequence of a fall [3]. Therefore, drugs that potentially affect cognition and may contribute to falls are of specific interest, such as drugs with potential anticholinergic side effects, cardiovascular drugs and drugs acting on the central nervous system [19, 20]. There was a specific focus on drugs with sedative effects, aiming at either dose reduction, changing regular use to use as needed, or, if possible, withdrawing the drug. Cardiovascular drugs may contribute to falls through adverse effects such as (orthostatic) hypotension or bradycardia. In addition, renal failure frequently occurs in frail elderly, particularly in relation to trauma and surgery, making dose adjustments or withdrawals of e.g. diuretics and renin-angiotensin inhibitors necessary.

If the patients were not already treated for osteoporosis and were considered having a sufficiently long life expectancy to benefit from medical treatment, a bone mineral density (BMD) measurement was performed, unfortunately after discharge for many patients. Treatment with calcium, vitamin D and bisphosphonates (orally or intravenously) was started in patients with low BMD.

In the CGC, frequent pain assessment was implemented by the use of a verbal rating scale. Paracetamol was used regularly in most patients and opioids on demand with morphine as the first choice and oxycodone as the second. As many patients were cognitively impaired and not able to communicate a need of opioids, analgesics were administered on a regular basis, with additional doses on request.

Most patients treated with opioids started treatment for constipation simultaneously. Frequency and amount of defecations were monitored in all patients with cognitive impairment. Lactulose was given to all and sodium picosulphate to many patients daily; in addition, bisacodyl was given either as needed or twice weekly. Doses were adjusted as appropriate.

### In the orthopaedic ward

The orthopaedic surgeons mainly focused at the surgical repair of the hip fracture, and treatment was performed in line with standard national guidelines. Other medical conditions including constipation and urinary tract infections were treated if patients had symptoms.

Analgesic treatment was based on paracetamol regularly and opioids as needed. There was no routine screening on vitamins, blood pressure, risk of falling or cognition.

### Variables

As described in a previous publication [12], information on previous and current diagnoses was included in the Charlson Comorbidity Index [21]. Baseline data and the patients’ drug regimen (at the time of the fracture and at discharge) were collected from the patients’ electronic records retrospectively. For preoperative risk classification, the Acute Physiology and Chronic Health Evaluation severity of disease classification system (APACHE II) was used, with scores ranging from 5 to 89, higher scores indicating a higher risk [22].

The drugs were classified according to the World Health Organization (WHO) Anatomical Therapeutic Classification (ATC) system [23]. There is no consensus on how to define polypharmacy [24], but for this study, we chose to define polypharmacy as using five or more drugs regularly, after having excluded drugs for topical use. If several medicines with the same active substance were used, this was counted as one drug. Drugs that were used regularly at admission to hospital and not at discharge were defined as withdrawn, whereas drugs not on the list at admission but used regularly at discharge were regarded as started during the hospital stay.

To classify drugs with anticholinergic properties, two different scoring systems were applied. The ARS scores drugs with anticholinergic properties in the range from 1 to 3, where a higher score indicates a more extensive anticholinergic effect [18]. Minor changes to ARS have been made by the authors based on a literature review, and the inhalation drugs tiotropium and ipratropium have been included with a score of 1 (Supplementary Table 1). In the present study, we also applied the scale in the review by Duran et al., where the drugs having low and high anticholinergic effects were given a score of 1 and 2, respectively [25] (Supplementary Table 2). The patient’s anticholinergic burden was defined by scoring the patient’s drugs according to each of these systems. The number of patients using one or more drugs as included by either scale in addition to the scales combined was also registered.

Instrumental activities of daily living (i-ADLs) before the fracture and at 4 months were assessed by the Nottingham Extended ADL Scale (NEAS; range 0 to 66 points; 66 best score) [26]. At 4 months after inclusion, mobility was assessed by the Short Physical Performance Battery (SPPB; range 0 to 12 points; 12 best score) [27], fear of falling was assessed by the Falls Efficacy Scale International—short form (FES-I-s; range 7–28 points; lower scores indicating less fear) [28] and quality of life was rated by the European Quality of Life Five-Dimension 3L Scale (EQ-5D-3L; range −0.594 to 1; higher scores indicating a better QoL) [29]. Follow-up assessment was performed by experienced health care professionals. Mobility was assessed at the hospital while data on the other outcomes were obtained by interviewing the patient or, if he or she was not able to respond, his or her next of kin.

### Statistical analysis

Statistical analyses were carried out using SPSS, Inc. (SPSS Statistics for Windows, version 20.0.; SPSS, Inc., Chicago). Values are reported as percentages or as means with standard deviations (SDs), as appropriate. For non-normally distributed variables, the Mann-Whitney *U* test was used to test for group differences. Group differences for categorical variables were tested by Pearson’s chi-square test. The exact unconditional z-pooled test was used for computing significance between proportions on small groups, with confidence coefficient = 0.9999. Due to multiple comparisons, two-sided *p* values <0.01 were regarded as statistically significant.

We carried out linear regression analyses with the primary and secondary outcomes SPPB, NEAS, FES-I-s and EQ-5D-3L scores as dependent variables, respectively, and the total number of changes in the drug treatment for each patient (the number of withdrawals plus the number of starts during the hospital stay in a patient) as covariate (model 1). Second, we adjusted for the pre-fracture NEAS score as a potential confounder (model 2). Third, we adjusted for the APACHE II score at admittance, and the total number of drugs used at admittance, as additional potential confounders (model 3). Three sets of analyses were carried out: in the OC group and the CGC group combined (i.e. all patients included in the study) and in the OC group and the CGC group separately. All analyses were adjusted for age, gender and fracture type, as was done in the main article [10].

## Results

A total of 1077 patients were screened for eligibility, of whom 397 were randomly assigned to receive either CGC (*n* = 198) or OC (*n* = 199). The most common reason for ineligibility was that the patients resided permanently in nursing homes (*n* = 250) or were <70 years (*n* = 154). Seven CGC patients and 12 OC patients died during the hospital stay, leading to 191 in the CGC group and 187 in the OC group (Fig. 1).

Baseline characteristics were similar in the groups with a mean age of 83 years, about 74% being females, and similar proportions of patients with a history of heart disease, stroke and cancer (Table 1).

**Table 1:** Baseline characteristics of 397 patients with hip fractures randomised to comprehensive geriatric care (CGC) in a geriatric ward or to usual orthopaedic care (OC) in an orthopaedic trauma ward

	CGC (*N* = 198)	OC (*N* = 199)
Age (years), mean (SD)	83.4 (5.4)	83.2 (6.4)
Gender (female), *n* (%)	145 (73.2)	148 (74.4)
Living alone, *n* (%)	115 (58.1)	124 (62.3)
Charlson Comorbidity Index (0–30), mean (SD)	2.3 (2.3)	2.3 (2.0)
Previous diagnoses, *n* (%)		
Heart disease	97 (49.0)	89 (44.7)
Stroke	49 (24.7)	57 (28.6)
Diabetes	23 (11.6)	28 (14.1)
Dementia	27 (13.6)	26 (13.1)
Cancer	53 (26.8)	43 (21.6)
Kidney disease	18 (9.1)	9 (4.5)
Fracture type, *n* (%)		
Femoral neck fracture	119 (60.1)	127 (63.8)
Extracapsular fracture	79 (39.9)	72 (36.1)
Length of stay (days), mean (SD)	12.6 (6.1)	11.0 (7.7)

As shown in Table 2, there were no statistically significant differences in drug use at admission. The mean number of drugs per patient was 3.8 (SD 2.8) in the CGC group and 3.9 (SD 2.8) in the OC group. Polypharmacy was found in 65 (32.8%) CGC patients and 79 (39.7%) OC patients (*p* = 0.16).

**Table 2:** Drugs used at admission and discharge from hospital and for patients randomised to comprehensive geriatric care (CGC) and orthopaedic care (OC)

	At admission			At discharge		
	*n* = 198	*n* = 199	*p* value	*n* = 191	*n* = 187	*p* value
Number of drugs used regularly per patient, mean (SD)	3.8 (2.8)	3.9 (2.8)	0.5	7.1 (2.8)	6.2 (3.0)	0.003
Number of drugs used as needed per patient, mean (SD)	0.32 (0.64)	0.56 (1.0)	0.0071	0.97 (1.1)	1.21 (1.17)	0.071
	*n* (%)	*N* (%)	*p* value	*n* (%)	*n* (%)	*p* value
Number of patients using ≥5 drugs	65 (32.8)	79 (39.7)	*0.16*	161 (84.3)	*132* (*70.6*)	*0.0015*
Alimentary tract and metabolism (ATC class A)	72 (36.4)	80 (40.2)	*0.44*	154 (80.6)	*117* (*62.6*)	*0.0002*
Constipation (A06A, A03FA, A03AX)	13 (6.6)	23 (11.6)	0.09	105 (55.0)	72 (38.5)	0.0014
Vitamins and mineral supplements (A11, A12)	37 (18.7)	32 (16.1)	0.5	102 (53.4)	53 (28.3)	0.0001
Calcium and calcium in combination with vitamin D (A12AA, A12AX)	19 (9.6)	19 (9.5)	1.0	83 (43.5)	15 (8.0)	0.0001
Blood and blood-forming organs (ATC class B)	108 (54.5)	110 (55.3)	*0.91*	171 (89.5)	*175* (*93.6*)	*0.16*
Cardiovascular system (ATC class C)	129 (65.2)	118 (22.1)	*0.23*	115 (60.2)	*113* (*60.4*)	*0.99*
Diuretics (C03)	42 (21.2)	41 (20.6)	0.90	33 (17.3)	42 (22.5)	0.262
Beta blockers (C07)	70 (35.4)	63 (31.7)	0.45	80 (41.9)	59 (31.6)	0.038
Calcium antagonists (C08)	25 (12.6)	27 (13.6)	0.81	11 (5.8)	24 (12.8)	0.018
Renin-angiotensin acting agents (C09)	61 (30.8)	44 (22.1)	0.05	44 (23.0)	43 (23.0)	1.0
Genito-urinary system and sex hormones (ATC class G)	10 (5.1)	14 (7.0)	0.42	12 (6.3)	13 (7.0)	0.84
Systemic hormonal preparations, excl. sex hormones, insulins (ATC class H)	30 (15.2)	44 (*22.1*)	*0.08*	30 (15.7)	*43* (*23.0*)	*0.07*
Anti-infectives for systemic use (ATC class J)	15 (7.6)	12 (6.0)	*0.55*	47 (24.6)	33 (17.6)	*0.10*
Antineoplastic and immune-modulating agents (ATC class L)	9 (4.5)	6 (3.0)	*0.49*	8 (4.2)	*6* (*3.2*)	*0.66*
Musculoskeletal system (ATC class M)	18 (9.1)	14 (7.0)	*0.47*	44 (23.0)	10 (5.3)	*0.0001*
Bisphosphonates (M05BA, M05BB)	12 (6.1)	7 (3.5)	0.24	41 (21.5)	6 (3.2)	0.0001
Nervous system (ATC class N), all drugs	77 (38.9)	83 (41.7)	*0.57*	177 (92.7)	*145* (*77.5*)	*0.0001*
Nervous system (ATC class N), analgesics excluded	55 (27.8)	64 (32.2)	0.36	39 (20.4)	67 (*35.8*)	0.001
Analgesics						
Opioids (ATC class N02A)	12 (6.1)	11 (5.5)	0.88	110 (57.6)	58 (31.0)	0.0001
Paracetamol (ATC class N02BE)	11 (5.6)	18 (9.0)	0.18	168 (88.0)	99 (52.9)	0.0001
Antipsychotics (N05A)	4 (2.0)	9 (4.5)	0.21	5 (2.6)	10 (5.3)	0.22
Antidepressants (N06A)	34 (17.2)	26 (13.1)	0.26	26 (13.6)	28 (15.0)	0.74
Anxiolytics (N05B)	6 (3.0)	9 (4.5)	0.49	0 (0.0)	10 (5.3)	0.0001
Hypnotics and sedatives (N05C)	25 (12.6)	38 (19.1)	0.08	14 (7.3)	34 (18.2)	0.0015
Respiratory system (ATC class R)	16 (8.1)	17 (8.5)	*0.92*	17 (8.9)	20 (10.7)	*0.57*
Sensory organs (ATC class S)	18 (9.1)	21 (10.6)	*0.65*	17 (8.9)	22 (11.8)	*0.37*
Drugs used as needed						
Alimentary tract and metabolism (ATC class A)	8 (4.0)	12 (6.0)	*0.39*	69 (36.1)	30 (16.0)	*0.0001*
Constipation (A06A, A03FA, A03AX)	4 (2.0)	9 (4.5)	*0.21*	65 (34.0)	24 (12.8)	0.0001
Cardiovascular system (ATC class C)	10 (5.1)	15 (7.5)	*0.31*	12 (6.3)	18 (9.6)	*0.24*
Nervous system	27 (13.6)	40 (20.1)	*0.09*	56 (29.3)	99 (52.9)	*0.0001*
Opioids (ATC class N02A)	8 (4.0)	13 (6.5)	0.29	14 (7.3)	67 (35.8)	0.0001
Paracetamol (ATC class N02BE)	7 (3.5)	10 (5.0)	0.48	14 (7.3)	47 (25.1)	0.0001

At discharge, the mean number of drugs used regularly was 7.1 (SD 2.8) in the CGC group and 6.2 (SD 3.0) in the OC group (*p* = 0.003). Polypharmacy was found in 161 (84.3%) CGC patients and 132 (70.5%) OC patients (*p* = 0.0015). A total of 209 withdrawals of drugs that had been used regularly were found in the CGC group, as compared to 82 in the OC group (*p* < 0.0001), with significantly more withdrawals of drugs with effect on the blood and blood-forming organs (ATC class B), cardiovascular system (ATC class C) and nervous system (ATC class N). In total, 844 and 526 new drugs were started in the CGC and OC groups, respectively (*p* < 0.0001). At discharge, significantly more patients in the CGC group used drugs with effect on the alimentary tract and metabolism (ATC class A), musculoskeletal system (ATC class M) and nervous system (ATC class N). When excluding analgesics at discharge, the number using drugs with effect on the nervous system was significantly higher in the OC group. Further details are given in Tables 2 and 3. The *r* values for the correlation between the length of stay and the total number of changes were 0.13 in the CGC group (*p* = 0.08) and 0.25 in the OC group (*p* = 0.001).

**Table 3:** Number of withdrawals and starts from hospital admission to discharge in the comprehensive geriatric care (CGC) and orthopaedic care (OC) groups

	No. of withdrawals			No. of starts		
	CGC	OC	*p* value	CGC	OC	*p* value
Gastrointestinal system and diabetes (ATC group A)	26	20	*0.53*	226	109	*<0.0001*
Constipation (A06A, A03FA, A03AX)	6	7	0.75	105	59	<0.0001
Vitamins (A11, A12)	6	8	0.96	94	33	<0.0001
Calcium and calcium/vitamin D combination (A12AA, A12AX)	0	3	0.08	64	2	<0.0001
Blood and blood-building organs (ATC group B)	29	10	*0.004*	190	176	*0.62*
Cardiovascular system (ATC group C)	86	20	*<0.0001*	47	31	*0.01*
Diuretics (C03)	15	4	0.01	9	13	0.60
Beta blockers (C07)	8	1	0.02	19	5	0.004
Calcium antagonists (C08)	14	5	0.04	0	3	0.08
Agents acting on the renin-angiotensin system (C09)	31	3	<0.001	14	5	0.04
Systemic infections (ATC group J)	4	2	*0.43*	44	27	*0.03*
Musculoskeletal system (ATC group M)	7	3	*0.21*	33	1	*<0.0001*
Bisphosphonates (M05BA, M05BB)	3	1	0.33	32	1	<0.0001
Nervous system (ATC group N)	50	21	*0.002*	281	168	*<0.0001*
Opioids (N02A)	6	3	0.33	104	54	<0.0001
Non-opioids (N02B)	0	4	0.04	157	86	<0.0001
Antipsychotics (N05A)	2	1	0.58	4	2	0.43
Anxiolytics (N05B)	5	1	0.11	0	4	0.04
Antidepressants (N06A)	14	1	0.001	8	7	0.83
Hypnotics and sedatives (N05C)	15	8	0.15	4	10	0.09
Others (ATC groups G, H, L P and R)	7	6	*0.81*	23	14	*0.15*
Sum	209	82	*<0.0001*	844	526	*<0.0001*

The anticholinergic burden measured by the ARS did not show any statistically significant differences between groups (Table 4). Using the classification by Duran et al. [25], the CGC group had a significantly higher anticholinergic burden for drugs used regularly and for all drugs used, whereas the OC group had non-significantly higher scores for drugs taken as needed. The variation in findings depending on the specific scale used was related to differences in the prescribing patterns of opioids between groups.

**Table 4:** Prescription of drugs with anticholinergic properties at hospital admission and discharge in the comprehensive care (GCG) group and in the usual orthopaedic care (OC) group. The Anticholinergic Risk Score (ARS) and the classification suggested by Duran et al. [25] have been used to score anticholinergic properties

	At admission			At discharge		
	CGC (*n* = 198)	OC (*n* = 199)	*p* value	CGC (*n* = 191)	OC (*n* = 187)	*p* value
Drugs used at a regular basis						
ARS score, mean (SD)	0.34 (0.87)	0.38 (0.88)	0.80	0.22 (0.59)	0.39 (0.88)	0.15
Duran score, mean (SD)	0.44 (0.82)	0.42 (0.82)	0.75	0.87 (1.1)	0.61 (0.99)	0.0001
No. of patients with ARS score >1, *n* (%)	38 (19.2)	39 (19.6)	0.94	31 (16.2)	39 (20.9)	0.25
No. of patients with Duran score >1, *n* (%)	40 (20.2)	40 (20.1)	1.0	112 (58.6)	60 (32.1)	0.0001
No. of patients with combined ARS + Duran >1, *n* (%)	65 (32.8)	64 (32.2)	0.91	134 (70.2)	79 (42.2)	0.0001
Drugs used regularly or as needed						
ARS score, mean (SD)	0.37 (0.90)	0.46 (0.98)	0.51	0.25 (0.62)	0.49 (0.97)	0.038
Duran score, mean (SD)	0.52 (0.94)	0.56 (1.06)	0.95	1.03 (0.92)	0.90 (1.21)	0.005
No. of patients with ARS score >1, *n* (%)	41 (20.7)	45 (22.6)	0.66	36 (18.8)	49 (26.2)	0.088
No. of patients with Duran score >1, *n* (%)	47 (23.7)	49 (24.6)	0.85	123 (64.4)	85 (45.5)	0.0003
No. of patients with combined ARS + Duran >1, *n* (%)	69 (34.8)	72 (36.2)	0.79	143 (74.9)	99 (52.9)	0.0001

At discharge, the regular use of analgesic treatment in the form of opioids and paracetamol was more frequently prescribed in the CGC, whereas paracetamol and opioids on request were prescribed more frequently in the OC group. Pharmacological treatment for constipation used regularly and/or as needed was significantly higher in the CGC group than in the OC group. Significantly more CGC than OC patients received treatment for osteoporosis with calcium/vitamin D and bisphosphonates at discharge, but even in the CGC group, only a minority of patients received pharmacological treatment for osteoporosis at discharge. The number of starts of other vitamins was also significantly higher in the CGC than in the OC group. On the other hand, significantly fewer patients used sedative and hypnotic drugs at discharge in the CGC group than in the OC group. Finally, antidepressants and cardiovascular drugs, in particular antihypertensives, were significantly more often discontinued in the CGC group than in the OC group during the hospital stay. Further details are given in Tables 2 and 3.

The association between the total number of changes in drug treatment during the hospital stay and the primary study outcome, mobility after 4 months (SPPB score), as well as the associations with the secondary outcomes NEAS score, FES-I-s score and EQ-5D-3L score are presented in Table 5. When adjusted for age, gender and fracture type only, the number of changes in drug treatment was negatively associated with the SPPB and NEAS score 4 months afterwards. These associations were found for all participants and, in addition, in the OC and CGC groups for SPPB and in the OC group for NEAS. However, when adjusting for the pre-fracture NEAS score, the APACHE II score, the Charlson Comorbidity Index and the number of drugs used at admission, these associations were no longer statistically significant. No significant associations were found with the outcomes fear of falling (FES-I-s score) and quality of life (EQ-5D-3L score).

**Table 5:** Linear regression with mobility, function, fear of falling and quality of life assessed by using respectively the Short Physical Performance Battery (SPPB), Nottingham Extended Activity of daily living Score (NEAS), Falls Efficacy Scale International—short form (FES-I-s) and European Quality of Life Five-Dimension 3L Scale (EQ-5D-3L) at 4 months as a dependent variable. All analyses were adjusted for age, gender and fracture type

	Effect of intervention (based on data from ref. [10])		Association with drug changes^a^					
			Model 1^b^		Model 2^c^		Model 3^d^	
	Difference^e^	*p* value	Coefficient	*p* value	Coefficient	*p* value	Coefficient	*p* value
SPPB (possible scoring range 0–12)								
All patients (*n* = 325)	*0.74* (*0.20* to *1.30*)	*0.010*	*−0.26* (*−0.41* to *−0.11*)	0.001	*−0.13* (*−0.25* to *0.00*)	0.050	−0.12 (−0.24 to 0.01)	0.069
GCG group (*n* = 165)			*−0.20* (*−0.40* to *−0.01*)	0.041	−0.06 (−0.22 to 0.11)	0.51	−0.06 (−0.3 to 0.10)	0.45
OC group (*n* = 160)			*−0.35* (*−0.58* to *−0.12*)	0.004	*−0.22* (*−0.42* to *−0.02*)	0.030	−0.19 (−0.4 to 0.01)	0.062
NEAS (possible scoring range 0–66)								
All patients (*n* = 330)	*6.17* (*2.57* to *9.78*)	*0.001*	*−1.12* (*−2.03* to *−0.21*)	0.016	−0.04 (−0.68 to 0.61)	0.91	−0.1 (−0.75 to 0.55)	0.76
GCG group (*n* = 167)			−0.82 (−1.97 to 0.33)	0.16	0.34 (−0.41 to 1.09)	0.37	0.25 (−0.50 to 1.00)	0.51
OC group (*n* = 163)			*−1.59* (*−3.07* to *−0.12*)	0.034	−0.6 (−1.70 to 0.51)	0.29	−0.57 (−1.70 to 0.52)	0.32
FES-I-s (possible scoring range 7–28)								
All patients (*n* = 298)	*−1.27* (*−2.27* to *−0.27*)	*0.013*	*0.04* (*−0.20* to *0.29*)	0.74	−0.07 (−0.30 to 0.16)	0.54	−0.11 (−0.34 to 0.12)	0.33
GCG group (*n* = 154)			*−0.01* (*−0.31* to *0.29*)	0.93	−0.11 (−0.39 to 0.17)	0.43	−0.15 (−0.43 to 0.13)	0.30
OC group (*n* = 144)			0.13 (−0.29 to 0.54)	0.55	−0.06 (−0.44 to 0.33)	0.77	−0.11 (−0.49 to 0.27)	0.57
EQ-5D-3L (possible scoring range −0.594 to 1)								
All patients (*n* = 305)	*0.08* (*0.01* to *0.15*)	*0.033*	−0.004 (−0.02 to 0.01)	0.65	0.006 (−0.01 to 0.02)	0.45	0.008 (−0.01 to 0.02)	0.31
GCG group (*n* = 157)			−0.003 (−0.03 to 0.02)	0.75	0.005 (−0.02 to 0.03)	0.63	0.005 (−0.02 to 0.03)	0.64
OC group (*n* = 148)			−0.003 (−0.03 to 0.02)	0.81	0.009 (−0.02 to 0.03)	0.46	0.013 (−0.01 to 0.04)	0.30

Associations are presented as regression coefficients with 95% confidence intervals and *p* values. Statistically significant values are presented in italics

^a^The total number of drug changes for each patient (the number of withdrawals plus the number of starts) during the hospital stay

^b^Adjusted for age, gender and fracture type

^c^Adjusted for age, gender, fracture type and pre-fracture NEAS score

^d^Adjusted for age, gender, fracture type, pre-fracture NEAS score, Acute Physiology and Chronic Health Evaluation severity of disease classification system (APACHE II) score, Charlson Comorbidity Index score and the number of drugs used at admission

^e^Difference in scores as an effect of being randomised to the GCG group as compared to the OC group

Additional analyses were carried out with the total number of drugs used at discharge and the number of antihypertensive drugs, opioids and psychotropic drugs at discharge with similar results as those reported above.

## Discussion

In the Trondheim Hip Fracture Trial, home-dwelling elderly patients with hip fractures were randomised to treatment in a geriatric ward (CGC group) or to an orthopaedic trauma ward (OC group). It has previously been shown that the CGC group had better mobility, function and quality of life during 12 months of follow-up after the hospitalisation and this treatment was cost-effective [10]. In the present study, we demonstrate that polypharmacy was more frequent in the CGC group at discharge, a difference that was mainly caused by the treatment of conditions related to the fracture, such as pain, constipation and osteoporosis, in addition to vitamins. Moreover, in the CGC group, more drugs were withdrawn, including cardiovascular drugs and CNS-active drugs, and more drugs were started during the hospital stay. The anticholinergic burden at discharge showed conflicting results between scales. There was a significant negative association between the total number of changes in drug treatment during the hospital stay and mobility and function 4 months later that were not statistically significant when adjusting for pre-fracture function, comorbidity and the number of drugs used at admittance. Changes in drug prescription could not explain the more beneficial outcome of the CGC group.

Patients in the CGC group underwent comprehensive geriatric care focusing on the assessment of all relevant comorbid disorders, review of drug regimen, screening for common conditions among geriatric patients and prevention of falls. This was not a part of the routine in an orthopaedic ward. The finding of an increased number of starts and withdrawals of drugs in the CGC group is in line with a previous study from our department [8] and demonstrated the extensive diagnostic work-up performed during the stay in a geriatric ward.

Drugs that are known to increase the risk of falls [20], such as antihypertensive drugs, antidepressants and sedatives/hypnotics, were withdrawn more frequently in the CGC group, a procedure that has previously been shown to prevent subsequent falls [4, 30]. Surprisingly, we found a significant negative association between the total number of changes in drug treatment during the hospital stay and mobility and function 4 months later in both groups. These associations were no longer statistically significant when adjusting for pre-fracture function, comorbidity and the number of drugs used at admittance. Length of stay was significantly correlated with the total number of changes in the OC group. Thus, the patients’ general health status at admittance was a clearly a confounding factor. It might be easier to stop treatment in a patient using numerous drugs at admittance due to multiple comorbidities, and it might be easier to start drug treatment in a patient with a multitude of diseases that could clearly have an impact on mobility and activities of daily living.

In addition to drug type, the Drug Burden Index (DBI) [31], which includes the administered doses of the drugs, would be of relevance to evaluate if treatment changes were of importance for beneficial impact of CGC. Furthermore, it is an open question whether we have been able to fully adjust for frailty and comorbidity. Unmeasured confounding may still exist, thereby making it impossible to explore the real effect of changes in the medication regimen with the study design employed here. Unfortunately, we are not able to perform these analyses as doses were lacking in the database and due to the retrospective design of the present study. Therefore, we cannot fully evaluate if our negative findings are related to methodological issues or reflect a lack of clinical importance.

The number of drugs used per patient at admission was lower than that in some other studies [3, 32], a difference that may be explained by the selection of home-dwelling patients in our study. When disease-specific treatment guidelines are applied to elderly patients with many disorders and symptoms, polypharmacy is often a consequence [33]. We chose a cut-off of five drugs, but this limit was rather arbitrarily chosen [24]. In our study, polypharmacy at discharge was found in most patients in both groups, and more so in the CGC group than in the OC group despite a particular focus on withdrawals of unnecessary drugs. In a study on the prevalence of polypharmacy and potential inappropriate drug use over a period of 15 years, Moriarty et al. showed that while the prevalence of polypharmacy increased, the odds for potential inappropriate prescribing declined during the same period [34]. Likewise, Belfrage et al., studying the quality of drug prescribing in patients with hip fractures, found that although there was a strong correlation between the number of drugs used and suboptimal drug prescribing, it was not possible to identify a cut-off for the number of drugs as a general indicator for the quality of drug prescribing [35]. Therefore, we argue that definitions of polypharmacy by using a fixed number of drugs is not a valid quality indicator for optimal prescribing and might also increase the risk of undertreatment of relevant disorders and symptoms.

Although the anticholinergic burden has been shown to increase the risk of dementia and predict adverse outcomes and in frail elderly [36, 37], intervention studies have failed to prove that withdrawal of anticholinergic drugs improves cognition [38]. In a study from our group published in 2005 [8], the use of drugs with anticholinergic properties was higher than that in the present study, indicating that prescribing in frail elderly may have improved during the last decade. In the present study, the anticholinergic burden was measured using two methods, showing different results. By using the ARS, there was no difference between the OC and the CGC groups. In contrast, when applying the classification by Duran et al. [25], the CGC group had higher anticholinergic burden, mainly related to the inclusion of opioids as a drug group with anticholinergic properties in the latter scale. A recent review concludes that the different scales available vary considerably in their content and that the scorings achieved by applying them in the clinical setting are most often not very predictive for the occurrence of anticholinergic adverse effects [39]. It is also known that the various measures of anticholinergic drug exposure could give different results when it comes to their association with other outcome variables, including mortality [40]. Thus, further research with validated methods is needed to evaluate the total anticholinergic burden in geriatric patients as well as to classify the inherent anticholinergic properties of opioid analgesics both as a group and for the individual agents [24, 35]. As an example, original studies point towards an anticholinergic effect of some opioid analgesics (specifically fentanyl, pethidine and tramadol) but not others, whereas opioids are most often included in the anticholinergic risk scales as a group [41, 42]. By including drug doses, such as in the DBI [31], the reliability of the scale could also possibly increase.

Treatment with analgesics other than paracetamol is a dilemma in a frail elderly person. NSAIDs should generally be avoided due to a risk of gastrointestinal, cardiovascular and renal adverse effects. However, opioids may increase the risk of falls and impair cognition. While the geriatricians preferred to prescribe opioids for regular use and anxiolytics and hypnotics as needed, orthopaedic surgeons prescribed opioids as needed and continued previous regular treatment with hypnotics and anxiolytics. Arguments for prescribing opioids regularly rather than intermittently as needed are that the risk of falls is highest shortly after initiating the treatment [43] and that in cognitively impaired patients, the risk of inadequate pain treatment is high if patients have to ask for opioids.

In accordance with other studies, only a minority of patients was treated for osteoporosis at admission to hospital [32]. In our study, treatment for osteoporosis was started more often in CGC patients than in OC patients. Nevertheless, even in the CGC group, most patients were not discharged with such treatment to a large extent because bone density measurements were performed after discharge from hospital. Unfortunately, we do not know how many patients started with treatment for osteoporosis after discharge.

This study has some weaknesses that need to be addressed but also some strengths. The strengths include the large sample and the randomised design. Among the weaknesses is the fact that data was collected retrospectively from patient records. It is also a major weakness that doses were not registered and the drug treatment appropriateness was not studied. Moreover, treatment both initiated and stopped during the hospital stay was not registered, as only drug use at admission and at discharge was recorded.

In conclusion, routines for review of drug regimens and drug prescribing were different in the CGC and the OC groups. We were, however, unable to demonstrate that the more beneficial outcome in the CGC group in the Trondheim Hip Fracture Trial [10] was associated with changes in drug therapy during the hospital stay. Although methodological limitations might explain this finding, a thorough diagnostic work-up by an interdisciplinary team specialised in geriatric medicine together with focus on early mobilisation and rehabilitation and discharge planning are held to be more important reasons for the more beneficial outcome in the GCG group.

## Electronic supplementary material

Supplementary Table 1 (PDF 443 kb)

Supplementary Table 2 (PDF 351 kb)

Supplementary Table 3 (PDF 232 kb)
