# Supplementary material for: Patterns of drug prescriptions in an orthogeriatric ward as compared to orthopaedic ward: results from the Trondheim Hip Fracture Trial—a randomised clinical trial
Source: Eur J Clin Pharmacol. 2017 May 26;73(8):937–47. doi: 10.1007/s00228-017-2263-x (PMC5508046; doi:10.1007/s00228-017-2263-x)
Supplement: Supplementary file 1 — (PDF 443 kb) [file 228_2017_2263_MOESM1_ESM.pdf]

Table 1. Anticholinergic Risk Scale, modified and updated from Rudolph et al [1]

Anticholinergic Risk Scale	
**ATC code**	**Name**
	**3 points**
A03AB02	Glycopyrronium bromide
A03BA01	Atropine
A03BA03	Hyoscyamine
A03BB01	Butylscopolamine
A04AD01	Scopolamine
G04BD04	Oxybutynin
M03BA02	Carisoprodol
N04AA02	Biperiden
N04AC01	Benzatropine
N05AA01	Chlorpromazine
N05AA02	Levomepromazine
N05AF03	Chlorprothixene
N05BB01	Hydroxyzine
N06AA02	Imipramine
N06AA04	Clomipramine
N06AA06	Trimipramine
N06AA09	Amitriptyline
N06AA12	Doxepin
R06AB02	Dexchlorpheniramine
R06AD01	Alimemazine
R06AD02	Promethazine
	**2 points**
A02BA01	Cimetidine
G04BD07	Tolterodine
G04BD08	Solifenacin
G04BD10	Darifenacin
G04BD11	Fesoterodine
M03BX01	Baclofen
N04BB01	Amantadine
N05AB02	Fluphenazine
N05AB03	Perphenazine
N05AB04	Prochlorperazine
N05AF01	Flupentixol
N05AF05	Zuclopenthixol
N05AH02	Clozapine
N05AH03	Olanzapine
N06AA10	Nortriptyline
R06AE03	Cyclizine

R06AE05	Meclozine
**1 point**	
ATC code	Name
A02BA02	Ranitidine
A02BA03	Famotidine
A03FA01	Metoclopramide
A07DA03	Loperamide
C01BA03	Disopyramide
N04BA02	Levodopa and decarboxylase inhibitor
N04 BA03	Levodopa, decarboxylase inhibitor, COMT inhibitor
N04BC04	Ropirinole
N04BC05	Pramipexole
N04BC06	Cabergoline
N04BC09	Rotigotine
N04BD01	Selegiline
N04BD02	Rasagiline
N04BX02	Entacapone
N05AD01	Haloperidol
N05AE04	Ziprasidone
N05AH04	Quetiapine
N05AL05	Amisulpride
N05AX08	Risperidone
N05AX12	Arpiprazole
N06AB05	Paroxetine
N06AX11	Mirtazapine
R03BB01	Ipratropium bromide
R03BB04	Tiotropium bromide
R06AE07	Cetirizine
R06AE09	Levocetirizine
R06AE27	Desloratidine
R06AX13	Loratidine

1 Rudolph JL, Salow MJ, Angelini MC, McGlinchey RE (2008) The anticholinergic risk scale and anticholinergic adverse effects in older persons. Archives of internal medicine 168 (5): 508-513 DOI 10.1001/archinternmed.2007.106
