# Supplementary material for: Patterns of drug prescriptions in an orthogeriatric ward as compared to orthopaedic ward: results from the Trondheim Hip Fracture Trial—a randomised clinical trial
Source: Eur J Clin Pharmacol. 2017 May 26;73(8):937–47. doi: 10.1007/s00228-017-2263-x (PMC5508046; doi:10.1007/s00228-017-2263-x)
Supplement: Supplementary file 2 — (PDF 351 kb) [file 228_2017_2263_MOESM2_ESM.pdf]

Supplementary table 2

Drugs classified as being high-potency anticholinergic drugs according to Duran et al [1] are given 2 points. Low-potency anticholinergic drugs according to the same review were given 1 point. Drugs marked with * were by Duran et al classified as strong, but having strong discrepancies between studies. After discussion with our pharmacological department they were included as low-potency drugs.

**2 points**	
**A03AA07**	Dicyclomine
**A03AB05**	Propantheline
**A03BA01**	Atropine
**A03BA03**	Hyoscyamine
**A03BA04**	Belladona alkaloids
**A04AD01**	Scopolamine (Hyoscine)
**G04BD01**	Emepronium
**G04BD02**	Flavoxate
**G04BD04**	Oxybutynin
**G04BD07**	Tolterodine
**G04BD10**	Darifenacin
**M03BX02**	Tizanidine
**N04AA01**	Trihexyphenidyl
**N04AA02**	Biperiden**
**N04AA04**	Procyclidine
**N04AA12**	Tropatepine
**N04AB02**	Orphenadrine
**N04AC01**	Benzatropine
**N05AA01**	Chlorpromazine
**N05AA02**	Levomepromazine
**N05AA04**	Acepromazine
**N05AB02**	Fluphenazine
**N05AC02**	Thioridazine
**N05AF04**	Thiothixene
**N05AH02**	Clozapine
**N05BB01**	Hydroxyzine
**N06AA0**	Clomipramine
**N06AA01**	Desipramine
**N06AA02**	Imipramine
**N06AA06**	Trimipramine
**N06AA09**	Amitriptyline
**N06AA10**	Nortriptyline
**N06AA11**	Protriptyline
**N06AA12**	Doxepin
**R03BB01**	Ipratropium
**R06AA02**	Diphenhydramine
**R06AA02**	Dimenhydrinate
**R06AA04**	Clemastine
**R06AA08**	Carbinoxamine
**R06AB01**	Brompheniramine
**R06AB02**	Dexchlorpheniramine
**R06AB04**	Chlorphenamin

**R06AC01**	Pyrilamine
**R06AD02**	Promethazine
**R06AE05**	Meclozine
**R06AX02**	Cyproheptadine
**S01FA05**	Homatropine
**1 point**	
**A02BA01**	Cimetidine
**A02BA02**	Ranitidine
**A03FA01**	Metocopramide*
**A03FA03**	Domperidone
**A07DA03**	Loperamide
**C01AA04**	Digitoxin
**C01AA05**	Digoxin*
**C01BA03**	Disopyramide
**C03CA01**	Furosemide*
**M01AB15**	Ketorolac
**M03BA02**	Karisoprodol*
**M03BA03**	Methocarbamol
**M03BX01**	Baclofen
**M03BX08**	Cyclobenzaprine
**M04AC01**	Colchicine*
**N02AA01**	Morphine
**N02AA05**	Oxycodone
**N02AB02**	Meperidine
**N02AB03**	Fentanyl
**N02AC04**	Propoxyphene
**N02AX02**	Tramadol
**N03AE01**	Clonazepam
**N03AF01**	Carbamazepine
**N03AF02**	Oxcarbazepine
**N04BB01**	Amantadine
**N04BC01**	Bromocriptine
**N04BX02**	Entacapone
**N05AA03**	Promazine
**N05AB03**	Perfenazine*
**N05AB04**	Prochlorperazine
**N05AB06**	Trifluoperazine*
**N05AD01**	Haloperidol
**N05AE02**	Molindone
**N05AG02**	Pimozide
**N05AH01**	Loxapine
**N05AH03**	Olanzapine
**N05AH04**	Quetiapine
**N05AN01**	Lithium
**N05AX08**	Risperidone
**N05BA01**	Diazepam
**N05BA02**	Chlordiazepoxide
**N05BA12**	Alprazolame
**N05CD05**	Triazolam

**N05CD07**	Temazepam
**N06AA16**	Dosulepin
**N06AB03**	Fluoxetine
**N06AB04**	Citalopram
**N06AB05**	Paroxetine
**N06AB08**	Fluvoxamine
**N06AF03**	Phenelzine
**N06AX05**	Trazodone
**N06AX06**	Nefazodone
**N06AX11**	Mirtazapine
**N07BC02**	Methadone
**R03DA04**	Theophylline
**R05DA03**	Hydrocodone
**R05DA04**	Codeine
**R06AD01**	Alimemazine
**R06AE07**	Cetirizine
**R06AX13**	Loratadine
**R06AX26**	Fexofenadine

** Biperiden were added after discussion with our pharmacological department according to published comments to the Duran review.

1. Duran CE, Azermai M, Vander Stichele RH: **Systematic review of anticholinergic risk scales in older adults**. *European journal of clinical pharmacology* 2013, **69**(7):1485-1496.
