# Supplementary material for: Patterns of drug prescriptions in an orthogeriatric ward as compared to orthopaedic ward: results from the Trondheim Hip Fracture Trial—a randomised clinical trial
Source: Eur J Clin Pharmacol. 2017 May 26;73(8):937–47. doi: 10.1007/s00228-017-2263-x (PMC5508046; doi:10.1007/s00228-017-2263-x)
Supplement: Supplementary file 3 — (PDF 232 kb) [file 228_2017_2263_MOESM3_ESM.pdf]

Supplementary Table 3. Assessment and treatment in the groups receiving comprehensive geriatric care (CGC) and orthopaedic care (OC)

**In-hospital assessment and treatment of hip fracture patients in The Trondheim Hip Fracture Trial**		
	**Standard treatment offered to both groups**	
	Preoperative intravenous fluid<br>Analgesia (preoperative femoral nerve block, regular paracetamol, opioids on demand)<br>Thromboembolic prophylaxis<br>Perioperative antibiotic prophylaxis<br>Surgery performed mostly in spinal anaesthesia	
	**Comprehensive geriatric care (CGC)**	**Orthopaedic care (OC)**
**Department**	Geriatric ward in Department of Internal Medicine	Trauma ward in Department of Orthopaedic Surgery
**Team members**	Geriatricians (also specialists in internal medicine), 0.13 per bed<br>Registered nurses, licensed practical nurses , 1.7 per bed<br>Physiotherapists, 0.13 per bed<br>Occupational therapists, 0.13 per bed	Orthopaedic surgeons, 0·11 per bed<br>Registered nurses, licensed practical nurses, 1.5 per bed<br>Physiotherapist, 0.09 per bed
**Non-pharmacological care**	Structured and systematic interdisciplinary care focusing on:<br>• Early mobilisation and initiation of rehabilitation<br>• Function<br>• Social situation<br>• Early discharge planning	Postoperative care focusing on<br>• Mobilisation<br>• Observed needs (i.e. pain, constipation)
**Medical assessment and treatment**	Systematic assessment and treatment of geriatric conditions and relevant comorbid disorders<br>Systematic assessment of mental health (cognition and affective disorders)<br>Review of drug regimen (indication, side-effects, interactions)<br>Systematic pain assessment by using verbal rating scale<br>Regular assessment of constipation, laxatives in patients using opioids,<br>Assessment of nutrition, vitamin D, B12 and folate<br>Screening of urinary tract infection<br>Screening of osteoporosis<br>Prevention of falls (blood pressure, orthostatism, drugs)	Assessment and treatment of the following conditions if symptoms<br>• Comorbid disorders<br>• Pain<br>• Constipation<br>• Urinary tract infections
